# The role of ECM-PIEZO1-axis-mediated mechanosensation in the central nervous system

**DOI:** 10.3389/fneur.2026.1881955

**Published:** 2026-08-03

**Authors:** Xiping Zhang, Yuchen Zhu, Siqi Song, Peijie Liu, Chenxu Liu, Hongguo Li, Yao Zhang, Chaojie Wang, Mengxiao Du, Dongmei Tang, Yushi Hu

**Affiliations:** School of Sports Medicine and Health, Sports Medicine Key Laboratory of Sichuan Province, Chengdu Sport University, Chengdu, China

**Keywords:** Alzheimer’s disease, blood–brain barrier, central nervous system, extracellular matrix, ischemic stroke, multiple sclerosis, neuroinflammation, PIEZO1

## Abstract

Central nervous system (CNS) diseases are characterized by high rates of disability and mortality, and their pathological progression is generally accompanied by abnormal remodeling of the extracellular matrix (ECM) composition and mechanical properties. The mechanosensitive cation channel PIEZO1 is widely expressed in neurons, microglia, astrocytes, oligodendrocytes, and endothelial cells of the CNS. It can precisely sense mechanical signals such as ECM stiffness, viscoelasticity, shear stress, and matrix protein cross-linking, and convert them into intracellular calcium signals and downstream biochemical reactions, thereby mediating the mechanobiological crosstalk between the ECM and cells and playing a key role in physiological processes such as neurodevelopment, synaptic plasticity, blood–brain barrier (BBB) homeostasis, neuroimmune regulation, and cell fate determination. In diseases such as Alzheimer’s disease (AD), ischemic stroke (IS), and multiple sclerosis (MS), abnormal stiffening or remodeling of the ECM can lead to excessive activation of PIEZO1, which, by regulating pathways such as nuclear factor-kappa B (NF-κB), Yes-associated protein/transcriptional coactivator with PDZ-binding motif (YAP/TAZ), calcium/calmodulin-dependent protein kinase II (CaMKII), and glutathione peroxidase 4 (GPX4), exacerbates neuroinflammation, BBB disruption, myelin destruction, neuronal ferroptosis, and defective axonal regeneration. This article systematically reviews the cellular expression profile of PIEZO1 in the CNS, the ECM-mediated activation mechanisms, and downstream signaling networks. It elucidates the regulatory role of the ECM-PIEZO1 axis in both the physiological functions and typical diseases of the CNS, aiming to provide a theoretical basis and new insights for mechanobiological research into the mechanisms and targeted therapies of CNS diseases.

## 1 Introduction

Due to their high rates of disability and mortality, neurological diseases are among the most pressing health concerns worldwide today. According to the World Health Organization, central nervous system (CNS) diseases include Alzheimer’s disease (AD), multiple sclerosis (MS), and ischemic stroke (IS), among others (1). These diseases not only significantly impair people’s health and social well-being but also impose a massive social and economic burden. Furthermore, due to the accelerating global aging of the population and increasing life expectancy, the prevalence of these diseases is rising sharply, resulting in annual losses exceeding one trillion dollars.

The extracellular matrix (ECM) is a highly dynamic, bioactive, and continuously remodeling component of the CNS microenvironment (2–4), a growing researches confirm that changes in the composition, structure, and function of the ECM are widespread across various CNS diseases, constituting one of their common pathological features (5–7). By influencing barrier function (8), immune responses (9), cell behavior (10), and neuroplasticity (10), the ECM contributes to disease progression, primarily through changes in ECM stiffness and the accumulation of abnormal ECM components.

The mechanosensitive cation channel PIEZO1 is expressed in neurons, microglia, oligodendrocytes, and endothelial cells in the CNS. It can detect changes such as variations in ECM stiffness and serves as a key downstream protein of the ECM in the CNS. As a critical protein mediating communication between the ECM and cells, it plays a pivotal role in CNS diseases through “mechanobiological” interactions and provides a potential target for future therapies targeting mechanical signaling pathways to treat CNS diseases.

This article focuses on reviewing the specific functions of the ECM-PIEZO1 axis in CNS diseases, examining changes in the ECM in CNS diseases, as well as the expression, downstream pathways, and roles of PIEZO1 in the CNS. Finally, it summarizes the applications of the ECM-PIEZO1 axis in common CNS diseases. Previous studies have largely focused on peripheral PIEZO1, whereas this paper systematically integrates the cross-regulation of ECM-PIEZO1 mechanical signaling across various CNS cell types and diseases, as well as how PIEZO1 acts as a mechanical decoder in the communication between CNS cells and the peripheral environment. This provides a new perspective for mechanobiology and lays a theoretical foundation for the development of treatments for CNS diseases based on mechanical sensing and regulation.

## 2 Structure and function of PIEZO1

PIEZO1 is a mechanosensitive cation channel protein whose three-dimensional structure is critical for its perception of mechanical signals from the ECM. A 2010 study first identified the PIEZO family of large transmembrane proteins and linked them to mechanosensation. As the channel body itself (11), PIEZO proteins are key components essential for the formation of mechanically activated cation channels (12); they can directly mediate the “force-electricity-calcium” transduction in response to mechanical stimuli on cells, without relying entirely on complex multiprotein complexes or being limited to indirect mechanisms via integrins and the cytoskeleton. The PIEZO1 family includes PIEZO1 and PIEZO2, which exhibit distinct functional differences. PIEZO1 is a non-perceptual mechanosensory channel that focuses on responding to mechanical forces generated within the cell itself and within tissues, rather than the primary perception of external mechanical stimuli. It responds to a wide range of mechanical stimuli, including membrane stretching, shear stress, and matrix stiffness, and is involved in developmental regulation and the maintenance of tissue homeostasis (13); In contrast, PIEZO2 is a sensory mechanosensitive channel that converts external pressure, touch, vibration, and traction into neural electrical signals. It is specialized for sensing cell indentation and plays a leading role in the perception of touch, proprioception, pain, and visceral mechanical states (14, 15). PIEZO1, as a key mechanosensitive ion channel, is widely expressed in the peripheral system and participates in the regulation of multi-organ homeostasis. It is highly expressed in peripheral tissues such as CD4 T lymphocytes (CD4 T) cells (16), temporomandibular joint condylar cartilage (17), bone tissue (18), intestinal epithelial cells (19), and cardiac fibroblasts (20). It plays a crucial role in peripheral physiological and pathological processes such as immune homeostasis, bone and cartilage metabolism, intestinal barrier maintenance, and fibrosis regulation. However, in the study of the CNS, PIEZO1 exhibits a more extensive and fundamental role. It is widely distributed in various cell types such as neurons, astrocytes, oligodendrocytes, and microglia (21). We will further elaborate on the role of PIEZO1 in CNS cells in the following text.

**Figure 1:**
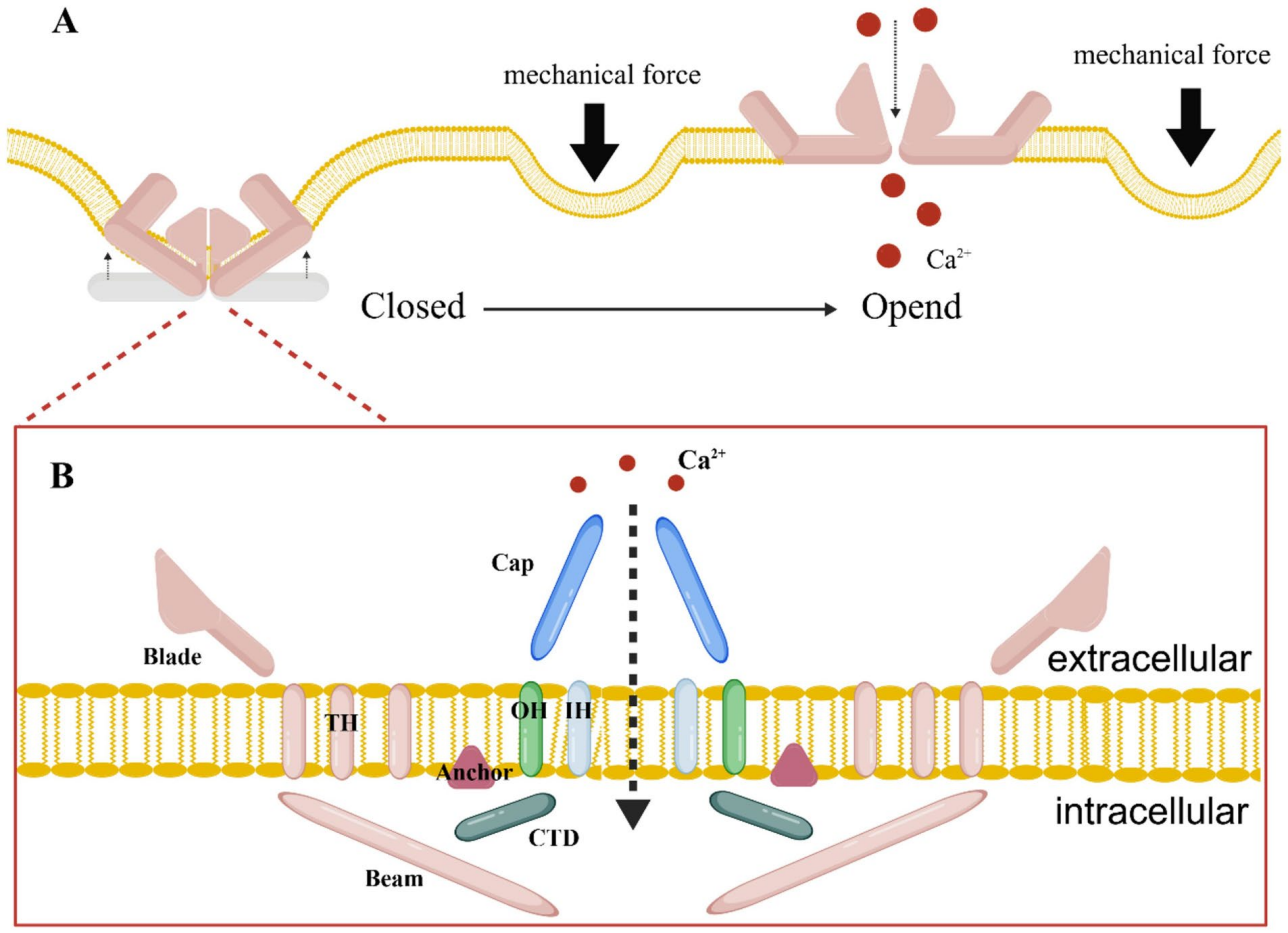
Schematic diagram of the PIEZO1 structure and its activation mechanism. **(A)** PIEZO1 is closed in the absence of mechanical stimulation; upon sensing mechanical force, membrane stretching activates the PIEZO1 channel, causing a calcium influx. **(B)** Schematic side view of a single subunit of the PIEZO1 mechanosensitive ion channel in a lipid bilayer, with key functional domains labeled: the extracellular cap domain (Cap), the intramembrane blade region (Blade) composed of transmembrane helices, the linker-anchor region (Anchor), the intracellular beam (Beam), the outer helix (OH) and inner helix (IH) that form the pore lining, and the cytoplasmic C-terminal domain (CTD). The black dashed line represents the central cation-permeable channel traversing the membrane. Created with BioGDP.com (107).

Regarding how PIEZO1 senses and transmits mechanical stimuli, a 2015 study using cryo-electron microscopy revealed the physical structure of the PIEZO1 channel for the first time, showing that PIEZO1 forms a massive transmembrane trimeric complex resembling a three-bladed propeller, with peripheral “blade” like structures and a central “cap”-like region (22). The peripheral structure of PIEZO1 likely acts like a giant lever arm to sense membrane tension and curvature, then transmits these mechanical changes to the central pore, thereby enabling the channel to switch between open and closed states. This is also why it can efficiently sense mechanical forces. Not only that, but PIEZO1 adopts a unique bowl-shaped conformation with a large membrane footprint, capable of inducing localized changes in cell membrane curvature. This distinctive spatial configuration makes it highly sensitive to membrane tension; when cells are subjected to mechanical stimuli such as matrix stiffness from the ECM, fluid shear stress, or tensile forces, membrane deformation can directly trigger a conformational shift in the channel, mediating the influx of calcium ions (23). It is worth noting that PIEZO1 exhibits conformational differences between its resting and activated states; these changes may be regulated by the cytoskeletal architecture, thereby influencing its efficiency in responding to mechanical signals. Furthermore, PIEZO1 acts as an active unit that not only responds to mechanical signals but also amplifies mechanical stimuli through structural changes (24). Figure 1 illustrates the structure of PIEZO1 and the mechanism of its activation.

Research has shown that the activation mechanism of PIEZO1 is dependent on matrix stiffness. In hard matrices, PIEZO1 is activated by cell membrane stretching, whereas in soft matrices—particularly in tissues such as the brain, which are low in stiffness and high in elasticity—PIEZO1 is not activated by significant stretching forces but rather by sensing energy dissipation (viscosity) in the matrix. Within the framework of biomechanics, viscosity—or the viscous modulus—is an intrinsic property of a material that determines its ability to irreversibly convert mechanical energy into thermal energy through internal friction during deformation; this conversion process is known as energy dissipation, the total amount of which is governed by viscosity; the dissipation rate characterizes the efficiency of energy dissipation per unit time and reflects the time-dependent response of viscoelastic materials to dynamic mechanical stimuli (25, 26). These three elements form a cascading logical chain that extends from material properties through the energy conversion process to dynamic signal intensity. The key mechanism by which PIEZO1 is activated in this context involves integrins; cells perceive the dissipative behavior of the matrix through adhesion complexes mediated by integrins (such as α5β1 under high shear stress and αvβ3/αvβ5 under low shear stress) (27), high dissipation rates promote dynamic turnover of adhesion plaques; force-dependent calcium signaling amplifies local membrane tension fluctuations, thereby triggering the opening of PIEZO1 channels (28). Experiments have confirmed that in soft hydrogels with a constant elastic modulus, simply increasing the viscoelastic modulus can effectively activate PIEZO1 and regulate the mechanosensitive behavior of mesenchymal stem cells (28). In summary, within a physiologically relevant range of stiffness (<1–5 kPa), viscoelasticity (dissipation rate) may serve as a more refined regulatory parameter than absolute stiffness; this explains why PIEZO1 plays a significant role in neural development, the Blood–Brain Barrier (BBB) and neuroinflammation.

In addition to sensing mechanical stimuli, PIEZO1 also serves as an indispensable mechanical signal receptor during the formation of the vascular network. PIEZO1 is expressed in developing vascular endothelial cells; its absence leads to marked abnormalities in vascular formation during the mid-embryonic stage in mice, ultimately resulting in embryonic death (29). This suggests that mechanical stimuli also play a crucial role in development, and that angiogenesis and vascular morphogenesis are significantly regulated by physical factors such as blood flow, pressure, and vessel wall tension. Furthermore, endothelial PIEZO1 does not merely passively respond to cerebral blood flow but participates in the mechanical feedback control of cerebral blood flow (30), laying the foundation for PIEZO1’s role in maintaining the BBB.

Moreover, PIEZO1 plays a crucial role in inflammation regulation. A 2019 study found that innate immunity is not driven solely by pathogens, inflammatory factors, and metabolic status; rather, the perception of periodic mechanical forces by immune cells is also a key input determining their function, and PIEZO1 is the central molecule in this process (31). In neurodegenerative diseases of the central nervous system, disruption of the BBB is often accompanied by the infiltration of immune cells, including macrophages. Concurrent with inflammation, there is also ECM deposition. Studies have demonstrated that PIEZO1 acts as a mechanical sensor for macrophage stiffness, and its activity regulates polarization responses (32), laying the groundwork for PIEZO1’s role in immunobiomechanics.

Finally, PIEZO1 also holds significance in peripheral tissues, playing a crucial role in bone metabolism, fibrosis regulation, and cell fate control. In 3D *in vitro* osteogenesis models, an increase in matrix hardness can also induce the activation of PIEZO1, thereby activating the adenosine monophosphate-activated protein kinase-unc-51-like autophagy activating kinase 1 (AMPK-ULK1) pathway and the expression of osteogenic-related genes (33). Moreover, in a rat alveolar bone defect model, sustained activation of PIEZO1 in stem cells significantly increased the enrichment of focal adhesions (FA) and cytoskeletal-related signaling pathways and upregulated the transcription levels of osteogenesis-related genes via a PIEZO1-integrin subunit α5 (ITGα5)-mediated mechanism (34); In a myocardial fibrosis model, simulated fibrotic ECM nanofiber matrices can induce the activation of PIEZO1, further activating the phosphatidylinositol 3-kinase-protein kinase B (PI3K-Akt) signaling pathway, causing the upregulation of matrix metalloproteinase 13 (MMP13) and thereby reducing the fibrotic area (35). Moreover, PIEZO1 may also regulate cell fate. The scar matrix left from previous injuries is not passively retained but can activate interstitial inflammation through PIEZO1, thereby promoting the occurrence of placental implantation spectrum diseases (36). Although this is a change that occurs in the periphery, PIEZO1 brings “the past mechanical history” into the current disease progression. In central degenerative diseases, glial scars are long-lasting and suggest that they can continuously rewrite cell behavior through PIEZO1.

## 3 ECM components of the central nervous system and their mechanical stimulation

The ECM in the CNS is a dynamic, bioactive scaffold that serves as the external environment essential for brain survival and is also the immediate environment through which PIEZO1 senses external mechanical stimuli. The ECM primarily consists of three major structures: the interstitial matrix, the basement membrane at the neurovascular and neuroepithelial junctions, and perineuronal nets (PNNs) (37). The interstitial matrix is composed of type I, III, and V collagen, fibronectin, proteoglycans, and glycosaminoglycans, while PNNs are composed of hyaluronic acid (HA), chondroitin sulfate proteoglycan (CSPG), and related proteoglycans, while the basement membrane consists of fibrous collagens—such as types I, III, and IV—as well as glycoproteins like laminin and fibronectin.

Under pathological conditions, changes in HA and CSPG in the ECM not only inhibit neural repair but also exacerbate the excitatory-inhibitory imbalance (38, 39) and neuroinflammation; furthermore, the ECM causes increased BBB permeability (8), thereby promoting disease progression. Primary human cerebral microvascular endothelial cells exhibit an inflammatory phenotype under a medium-stiffness matrix (approximately 30 kPa), but moderate flow shear stress can alleviate this phenomenon (40). Changes in the mechanical properties of the ECM not only reflect the characteristics of neurological diseases but also play a profound role in maintaining the physiological functions and pathological evolution of the CNS by regulating key processes such as synaptic plasticity, axonal regeneration, neurodevelopment, myelination, neuroinflammatory responses, microenvironmental homeostasis, and pathological remodeling in various neurodegenerative diseases, such as AD and MS.

Current research has demonstrated that the ECM can influence PIEZO1 function through various mechanisms, thereby serving as a mechanical signal that triggers PIEZO1 activation in the CNS. The following sections will summarize this ECM-mediated mechanical signaling from several perspectives.

The most important mechanical signal for activating PIEZO1 is the increase in ECM stiffness. In the neuronal model induced by hypoxia-ischemia (HI), the contents of type I and type III collagen increase, accompanied by an increase in PIEZO1 expression and channel activation (41–43). A harder ECM can activate PIEZO1, causing Ca^2+^ influx, leading to intracellular Ca^2+^ imbalance and elevated levels of reactive oxygen species (ROS), ultimately resulting in endoplasmic reticulum stress, oxidative stress, ferroptosis or osteogenic differentiation.

In addition to static stiffness, the viscoelasticity of the ECM has also been shown to influence the function of PIEZO1. Researchers investigated this using a hydrogel capable of independently modulating its elasticity and viscosity; in this context, PIEZO1 was more likely to be activated by viscoelasticity on softer matrices, further mediating mechanical signaling in stem cells and changes in gene expression profiles (28), indicating that PIEZO1 can sense not only static but also dynamic stimuli.

Different proteins in the ECM play important roles in the perception of mechanical stimuli by PIEZO1. In a high shear stress environment, fibronectin can increase the activity of PIEZO1 through α5β1 and αvβ3 integrins; while in a low shear stress environment, type I collagen, type IV collagen, and laminin can enhance the sensing ability of PIEZO1 to external forces via αvβ3 or αvβ5 integrins (27). Meanwhile, specific laminin isoforms—exemplified by laminin-411, which localizes to the perivascular niche during myelination—act as distinct mechanical stimuli that specifically trigger PIEZO1 activation in oligodendrocytes (44). This indicates that the ECM not only provides mechanical support but also sends precise signals through certain ligand-integrin-PIEZO1 pathways.

Finally, PIEZO1 can also be activated by ECM remodeling and cross-linking. In ischemic encephalopathy, increased lysyl oxidase (LOX) activity leads to elevated levels of its downstream substrates, collagen I and III, which in turn cause ECM remodeling and stiffening, thereby activating PIEZO1. This suggests that biochemical modifications of the ECM can influence its mechanical properties and indirectly regulate PIEZO1.

In summary, the ECM generates mechanical force signals capable of activating PIEZO1 within the CNS through variations in hardness, viscoelasticity, the presence of certain proteins, and cross-linking patterns. These mechanical force signals can directly influence PIEZO1 channel activation or exert their effects via mediators such as integrins, playing a crucial role in neural plasticity, development, and various CNS disorders. Potential upstream factors in the ECM that may activate PIEZO1 are shown in Table 1.

**Table 1:** Potential upstream factors of PIEZO1 activation within the ECM.

Trigger	Substances that undergo change	Final results
MS	accumulation of aggrecan, versican, and neurocan (108)	Inhibition of axon growth and myelin regeneration
	Deposition of CSPGs (109)	Enhanced inflammatory response
	Circulating fibrinogen (89) and fibronectin deposit around endothelial cells.	BBB disruption and immune cell infiltration (6)
	deposition of fibrinogen (89) and fibrin (110)	activate microglia and promote inflammation (89)
	deposition of HA (111)	Inhibition of OPCs maturation and promotion of demyelination (111)
AD	accumulation of CSPGs	Limited plasticity
	deposition of Aβ plaques (73)	Activates microglia and reduces the release of pro-inflammatory factors (51)
	degradation of PNNs (70)	deposition of Aβ plaques
IS	formation of glial scars	Inhibits axonal and myelin repair; promotes inflammation
HIBD	deposition of type I and type III collagen (42)	ferroptosis (42)
Bionic 3D Matrix Stiffness (112)	elevation of matrix stiffness	promote glycolysis
High shear stress (27)	Fibronectin, α5β1 and αvβ3 integrins	Enhance the response of PIEZO1 to shear stress
Low shear stress (27)	Collagen I and IV, laminin, and αvβ3 and αvβ5 integrins	Increase the mechanical sensitivity of PIEZO1

OPCs, oligodendrocyte precursor cells; Aβ, amyloid β-protein.

## 4 Integration of the ECM-triggered PIEZO1 downstream signaling pathway

The downstream pathways of PIEZO1 triggered by ECM primarily include integrins, adenosine triphosphate (ATP), and the Nuclear Factor-kappa B (NF-κB) pathway, among others. Their specific effects are mainly reflected in alterations to PIEZO1’s sensing of mechanical stimuli, myelination, ferroptosis, neural plasticity, and inflammation.

In CNS and related research, the ECM, integrins, and PIEZO1 constitute a sophisticated mechanical signal sensing and transduction system. In the ECM, fibronectin, collagen, and laminin can modulate PIEZO1’s sensitivity to mechanical stimuli through various integrins, such as integrin α5β1, αvβ3, and αvβ5. Under high shear stress conditions, fibronectin can increase PIEZO1’s sensitivity to mechanical stimuli via integrins α5β1 and αvβ3, whereas in low shear stress environments, type I collagen, type IV collagen, and laminin can regulate PIEZO1’s activation capacity via integrins αvβ3 and αvβ5 (27). Furthermore, as the primary molecules linking cells to the ECM, integrins trigger the formation of integrin-adhesion complexes upon binding to the ECM (45), thereby translating extracellular mechanical cues into intracellular signals. This reveals that integrins not only enhance PIEZO1’s mechanical sensitivity but may also functionally contribute to mechanical transduction.

The most important role of PIEZO1 in oligodendrocytes is the formation and protection of myelin. Pharmacological activation of PIEZO1 induces demyelination and neuronal damage, whereas its antagonist Grammostola spatulata mechanotoxin 4 (GsMTx4) exhibits neuroprotective effects and prevents chemically induced demyelinating lesions, suggesting that PIEZO1 activation inhibits the developmental behavior of oligodendrocytes. This phenomenon suggests that under pathological or physiological conditions, when the ECM undergoes stiffening due to injury, aging, or disease, the sustained activation of PIEZO1 may inhibit the normal function of oligodendrocytes, thereby disrupting myelin formation or repair. However, in the brain tissue of patients with MS, PIEZO1 expression in white matter regions was significantly lower than in healthy controls, and there was no apparent difference between the lesion areas and the apparently normal white matter (46). This seemingly contradictory result actually supports the dual role of PIEZO1 in myelin homeostasis: on the one hand, moderate PIEZO1 signaling may be involved in maintaining oligodendrocytes’ ability to sense the mechanical microenvironment; on the other hand, under chronic demyelination conditions, downregulation of PIEZO1 expression may reflect functional exhaustion or compensatory suppression, further impairing the potential for myelin regeneration. Even so, the specific mechanisms underlying the interaction between PIEZO1 and myelin have not yet been fully elucidated. In peripheral tissues, PIEZO1 can regulate axon regeneration through the calcium/calmodulin-dependent protein kinase II-focal adhesion kinase-actin (Ca^2+^-CaMKII-FAK-actin) signaling cascade to regulate axonal regeneration. Although this pathway has not been described in the CNS, given that FAK and actin cytoskeleton dynamics are equally critical during myelination in the CNS, this pathway likely plays a similar role in central myelination; In Schwann cells, PIEZO1 is activated by mechanical stimulation of the ECM and subsequently regulates myelination by modulating the activity of the Yes-associated protein/transcriptional coactivator with PDZ-binding motif (YAP/TAZ) complex (47), YAP/TAZ are key effectors of the Hippo signaling pathway, and their activity is influenced by cytoskeletal tension and mechanical forces, suggesting that PIEZO1 may also regulate myelination through the YAP/TAZ cytoskeletal axis. During the progression of CNS diseases, alterations in the mechanical properties of the ECM may represent a potential upstream mechanism triggering neuronal ferroptosis. In traumatic brain injury, LOX increases collagen I/III components, promotes ECM remodeling, and enhances matrix stiffness; this mechanical signal is specifically detected by the mechanosensitive channel PIEZO1, thereby triggering a calcium influx that leads to the inhibition of glutathione peroxidase 4 (GPX4) activity, the accumulation of lipid peroxidation, and an imbalance in iron metabolism, ultimately driving neuronal programmed cell death. Notably, this process can be reversed by ferroptosis-specific inhibitors, and pharmacological inhibition of LOX or PIEZO1 not only significantly reduces lipid peroxidation-induced damage and neuronal loss in key brain regions such as the hippocampus but also effectively improves learning and memory impairments. At the mechanistic level, this pathway is integrated into the “mechanical force-PIEZO1-Ca^2+^-iron/lipid metabolism” axis, highlighting the role of mechanical stress as an upstream regulatory hub for ferroptosis and incorporating ECM remodeling, metabolic reprogramming, and neuroinflammation regulation into a unified signaling network (48). In addition to ferroptosis, PIEZO1 is also involved in neuronal apoptosis. Intracranial hypertension resulting from subarachnoid hemorrhage induces PIEZO1 expression, which leads to neuronal apoptosis and neurological dysfunction through activation of the Hippo signaling pathway.

In the CNS, changes in the physical and biochemical properties of the ECM act as upstream mechanical signals that can trigger a series of downstream signaling pathways by activating the mechanosensitive PIEZO1 channel in glial cells, such as astrocytes, thereby regulating neuroplasticity, neurogenesis, and cognitive function. In AD models, astrocytes surrounding mechanically rigid amyloid plaques significantly upregulate PIEZO1 expression (49). Upon activation, PIEZO1 mediates calcium influx and further promotes ATP release-a process critical for adult neurogenesis in the hippocampus. Astrocyte-specific deletion of PIEZO1 leads to a significant reduction in hippocampal volume and whole-brain weight, impaired hippocampal long-term potentiation (LTP), and deficits in learning and memory behavior, as well as severe impairment of adult neurogenesis; however, exogenous supplementation with ATP can reverse this process (50). This pathway indicates that ATP release is a key effector molecule downstream of PIEZO1 and is crucial for neural plasticity, neurogenesis, and cognitive function.

Regarding inflammatory pathways, in microglia, the calcium influx mediated by PIEZO1 activation specifically inhibits the NF-κB pathway, thereby downregulating the secretion of tumor necrosis factor-*α* (TNF-α) and interleukin-6 (IL-6), and alleviates pro-inflammatory activation of microglia. This effect is dependent on calcium signaling and can be blocked by PIEZO-specific small interfering RNA (siRNA) or the PIEZO1 inhibitor ruthenium red (51). Notably, in patients with drug-resistant temporal lobe epilepsy and in animal models, PIEZO1 expression levels in the hippocampus are positively correlated with inflammatory markers such as TNF-*α*, interleukin-1β (IL-1β), and NF-κB; furthermore, recombinant human TNF-α can upregulate PIEZO1 expression in astrocytes (52). This suggests a bidirectional regulatory relationship between PIEZO1 and inflammatory pathways, and that its role varies across different cell types.

Therefore, when PIEZO1 is activated by the ECM in the CNS, it not only collaborates with other integrins—such as ITGα5 and αvβ3-to sense mechanical signals from the ECM, thereby altering cell shape and function, but also mediates myelination, ferrocytosis, neuronal apoptosis, and neuroplasticity; it can even inhibit NF-κB-mediated inflammatory responses through a calcium-dependent mechanism. Although some ECM-triggered PIEZO1 pathways have been identified, the entire signaling network within the CNS requires further investigation. Table 2 lists common PIEZO1 downstream pathways in the CNS.

**Table 2:** Common downstream pathways of PIEZO1 activation in the CNS.

Downstream distribution channels	Result
Inhibits the NF-κB inflammatory signaling pathway (51)	Anti-inflammatory
Activates the RHOA/ROCK pathway; increased expression of ITGα5 and TNS1 protein further activates PIEZO1 (34)	Stimulates osteogenic differentiation and promotes alveolar bone regeneration
Activates PiCS Channel (113)	Cytoskeletal stiffening, adaptation to matrix stiffness, promotion of fibrosis, cell migration
Activates YAP/TAZ signaling pathway (47)	Inhibits myelination
Microglia activation (114)	Enhance phagocytic capacity and reduce Aβ deposition
Activates the CaMKII-Nrf2 axis (85)	Exacerbate oxidative stress and inflammatory responses in the endothelium
Activates the Nrf2/HO-1/NQO1 signaling pathway (68)	Exacerbate oxidative stress and inflammatory responses in the endothelium
Inhibition of the TGFβ/SMAD signaling pathway (99)	Inhibits the activity of Treg and promotes inflammation
Reduced GPX4 expression leads to ferroptosis	Learning and memory impairments and impaired synaptic plasticity
Triggers Ca^2+^ influx, activates the AP-1 transcription factor, and upregulates Edn1 expression (31)	Macrophages express pro-inflammatory molecules, and inflammation occurs
Calcium ions flow into the cell, thereby triggering the release of calcium ions from the endoplasmic reticulum (102)	Demyelination
Activates the CaMKII-Nos-PKG pathway (103)	Inhibit axonal regeneration

RHOA/ROCK, Ras homolog family member A/Rho-associated coiled-coil-containing protein kinase; TNS1, tensin 1; PiCS, Piezo1-induced Cytoskeletal Stiffening; CaMKII-Nrf2, calcium/calmodulin-dependent protein kinase II–nuclear factor erythroid 2–related factor 2; Nrf2/HO-1/NQO1, nuclear factor erythroid 2–related factor 2/heme oxygenase-1/NAD(P)H quinone dehydrogenase 1; TGFβ/SMAD, transforming growth factor-β/Sma- and Mad-related protein; Treg, regulatory T cells; AP-1, activator protein-1; Edn1, Endothelin-1; CaMKII-Nos-PKG, calcium/calmodulin-dependent protein kinase II–nitric oxide synthase–protein kinase G.

## 5 The role of PIEZO1 in central nervous system cells

The expression of PIEZO1 is not entirely consistent across different CNS cell types, and its function may also vary; changes over time may also lead to alterations in the function of PIEZO1.

### 5.1 PIEZO1 in neurons

Neurons, as the key cells for the transmission of electrical signals, are the most numerous in the CNS. They are also involved in various complex physiological processes, including the integration of mechanochemical signals, neurogenesis, axon guidance and regeneration, the regulation of synaptic plasticity, and interactions with glial cells. Studies have shown that substrates with a stiffness of 200 kPa promote axonal regeneration, whereas softer substrates (e.g., 0.1–1 kPa, which is close to that of physiological brain tissue) may inhibit axonal regeneration (53). Softer substrates may alter membrane curvature and FA mechanics, thereby activating PIEZO1 (54), which in turn inhibits axonal growth via the Ca^2+^-CaMKII-FAK-actin pathway, however, this phenomenon has only been observed in the peripheral nervous system; In contrast, within the CNS, ECM cross-linking-mediated matrix stiffening can remodel the actin cytoskeleton, thereby activating YAP-dependent gene transcription programs, which ultimately significantly enhance the potential for neuronal axonal regeneration (55). Furthermore, a reduction in PIEZO1 in the cerebral cortex lowers the levels of the distant chemical guidance factors semaphorin 3A (Sema3A) and slit guidance ligand 1 (Slit1), causing axons to misdirect and softening the region (56), indicating that the bidirectional regulation of extracellular matrix stiffness by PIEZO1 is crucial for axon development, with PIEZO1 playing a key role in this regulation.

In models of CNS injury, abnormal activation of PIEZO1 has been shown to be closely associated with neuronal apoptosis. Under conditions of elevated intracranial pressure following subarachnoid hemorrhage, PIEZO1 expression is upregulated and promotes neuronal apoptosis by activating the Hippo signaling pathway; however, PIEZO1 inhibition significantly reduces neuronal apoptosis and improves neurological deficits, and this protective effect can be reversed by Hippo pathway agonists (57); Similarly, in a cerebral ischemia–reperfusion model, the PIEZO1 agonist Yoda1 exacerbated neuronal calcium overload, activation of calcium-activated neutral protease (calpain), and apoptosis, whereas its inhibitor GsMTx4 exerted a neuroprotective effect (58).

In summary, PIEZO1 not only acts as a sensor of the mechanical microenvironment to bidirectionally regulate axon development in central neurons, but also mediates neuronal damage and death via calcium signaling pathways under pathological conditions, highlighting its potential value as a target for neural repair interventions.

### 5.2 PIEZO1 in microglia

PIEZO1 is widely expressed in microglia; its activation regulates various key processes associated with neurodegenerative diseases. It forms a dynamic “interaction network” with the ECM, maintaining homeostasis under physiological conditions but becoming dysregulated during aging or disease. This dysregulation manifests as abnormal mechanical transduction, elevated protease activity, and sustained release of pro-inflammatory factors (37), ultimately compromising ECM integrity and impairing neurovascular and synaptic function. Specifically, PIEZO1 activation induces intracellular calcium influx and inhibits the NF-κB inflammatory signaling pathway, thereby significantly downregulating the production of the pro-inflammatory cytokines TNF-*α* and IL-6 in response to lipopolysaccharide (LPS) stimulation. This effect can be blocked by PIEZO1-specific siRNA, BAPTA-AM (a cell-permeable calcium chelator), or the channel inhibitor ruthenium red, indicating that PIEZO1-mediated calcium signaling is a key mechanism regulating the pro-inflammatory phenotype of microglia (51).

Further studies have shown that PIEZO1 deficiency in the tumor microenvironment inhibits the differentiation of T cells into the antitumor T helper 9 (Th9) cell subset. This is due to the inhibition of the Sirtuin 3-succinate dehydrogenase complex flavoprotein subunit A -oxidative phosphorylation (SIRT3-SDHA-OXPHOS) pathway and the activation of hypoxia-inducible factor 1α (HIF1α) signaling (59), suggesting that PIEZO1 influences cell differentiation by modulating redox metabolism. In the nervous system microenvironment, similar mechanisms may also occur in microglia or neural progenitor cells. When PIEZO1 is activated, it causes an increase in cytoplasmic calcium ions, which in turn affects energy metabolism and mitochondrial function, thereby determining whether cells enter proliferation, differentiation, or apoptosis.

Although PIEZO1 exerts an anti-inflammatory effect on microglia, most existing studies have focused on the post-onset stage of AD. Future research should clarify the dynamic effects of PIEZO1 on the polarization state of microglia at different stages of the disease, as well as whether its role is consistent across different diseases. Furthermore, it is necessary to verify whether PIEZO1 regulates the fate of microglia or neural progenitor cells via the calcium-mitochondrial axis.

### 5.3 PIEZO1 in astrocytes

Astrocytes in the CNS are not only structural and metabolic support cells, but also key regulators that dynamically sense changes in the microenvironment (including chemical, inflammatory, and mechanical signals), modulate neuroimmune interactions, and influence neural plasticity and regeneration. Studies (50) have shown that astrocytes express PIEZO1 and can use it to sense mechanical stimuli in the local microenvironment, converting these stimuli into intracellular calcium signals that trigger ATP release, thereby maintaining the connection between neurons and glial cells. However, the absence of PIEZO1 leads to a reduction in hippocampal volume and brain weight, severely impairs adult neurogenesis, and eliminates the ATP-dependent enhancement of neural stem cell proliferation *in vitro*; at the same time, overexpression of PIEZO1 enhances LTP and learning and memory abilities, indicating that it plays a positive role in cognitive function (60). Following IS, downregulation of PIEZO1 expression in astrocytes activates the Wnt family member 7B /Ca^2+^ (Wnt7b/Ca) non-canonical signaling pathway, promoting cytoskeletal reorganization and reducing glial scar stiffness, thereby facilitating nerve regeneration and motor function recovery; it is a key factor in regulating the formation of the physical barrier of glial scars (61). In terms of anti-inflammation, unlike microglia, PIEZO1 expression is positively correlated with the pro-inflammatory factors TNF-α, IL-1β, and NF-κB activity; this is due to TNF-α upregulating PIEZO1 expression in turn, rather than PIEZO1 itself driving inflammation (52).

Not only that, but astrocytes can also determine cell fate through PIEZO1. Upon activation of PIEZO1 in astrocytes, Ca^2+^ influx and ATP secretion occur; the latter acts as a paracrine factor that induces neural stem cells to transition from a quiescent state to a proliferative state and promotes their further development into the neuronal lineage (50). In astrocytes of the optic nerve head (ONH), PIEZO1 deficiency may lead to reduced nuclear localization of YAP and downregulation of downstream cell cycle-related factors such as Cyclin D1 and c-My, resulting in cell cycle arrest at the quiescent G0 phase and the DNA-synthesizing prephase G1 (G0/G1) phase, thereby regulating astrocyte proliferation (62).

Overall, ECM-PIEZO1 is a neuroimmunomodulator that plays a crucial role in sensing and responding to subtle changes. Under normal conditions, it is essential for maintaining normal tissue cell function and promoting the release of ATP by astrocytes, thereby supporting neurogenesis and synaptic plasticity (50); However, under pathological conditions, changes in the elastic modulus of the ECM or increased levels of inflammatory factors may lead to its excessive activation, resulting in adverse consequences such as neuroinflammation, ferrocytosis, and the repositioning of immune cells.

### 5.4 PIEZO1 in oligodendrocytes

PIEZO1 is specifically expressed in oligodendrocytes (21, 63), and its function is closely associated with myelin homeostasis and pathological stress responses in the CNS. In acute injury models such as intracerebral hemorrhage, abnormal activation of PIEZO1 can exacerbate endoplasmic reticulum stress in oligodendrocytes. Through the protein kinase RNA-like endoplasmic reticulum kinase-activating transcription factor 4-C/EBP homologous protein (PERK-ATF4-CHOP) and inositol-requiring enzyme 1α (IRE1) signaling pathways, promoting apoptosis and leading to myelin loss and neurological dysfunction. Furthermore, in sepsis-associated encephalopathy, PIEZO1 activation can also induce ferroptosis in oligodendrocytes via the p38 mitogen-activated protein kinase (p38 MAPK) pathway mediated by microglia (64).

Taking a broader view, PIEZO1 not only acts on oligodendrocytes but is also associated with OPCs. As age increases, the stiffness of the ECM gradually rises, leading to age-related functional loss in OPCs; however, *in vivo* inhibition of PIEZO1 can counteract the effects of mechanical signals, allowing OPCs to remain active in the aging CNS (65).

In summary, the effects of PIEZO1 on oligodendrocytes are not limited to maintaining myelin homeostasis; it also plays a role in the past and future of oligodendrocytes, regulates the differentiation of progenitor cells, and may, to some extent, interact with other CNS cells to determine the fate of oligodendrocytes.

### 5.5 PIEZO1 in endothelial cells

In the CNS, although endothelial cells are not included, the dynamic stability of the BBB requires close coordination among endothelial cells of the neurovascular unit (NVU), astrocytes, pericytes, microglia (66), as well as the involvement of the mechanical properties of the ECM and the mechanoreceptors present on its surface. The dynamic equilibrium of the BBB plays a crucial role in maintaining the stability of the intracerebral environment and can lead to the onset of various CNS diseases. Previous studies have shown that PIEZO1 acts as a bridge mediating mechanical signals from the ECM to BBB functional homeostasis. It plays a crucial role in detecting blood flow shear stress, changes in tissue stiffness, or ECM remodeling, generating mechanical stimuli that are converted into intracellular Ca^2+^ signals, thereby regulating BBB permeability and integrity.

Under normal conditions, PIEZO1 regulates cerebral blood flow by sensing hemodynamic forces (such as shear stress); in this process, it depolarizes endothelial cells to prevent hyperemia and promote a return of blood flow to baseline levels, thereby maintaining neurovascular coupling and BBB integrity (30). Furthermore, PIEZO1 plays a crucial role in meningeal lymphangiogenesis and cerebrospinal fluid flow; its deficiency leads to ventricular dilation and hydrocephalus (67), which in turn affects BBB stability. At the same time, ECM components (such as fibronectin, laminin, and type I and IV collagen) can alter PIEZO1’s sensitivity to different shear stresses via integrin pathways (e.g., α5β1,αvβ3,αvβ5, etc.) to alter PIEZO1’s sensitivity to different shear forces, indicating that the ECM-PIEZO1 complex functions as a variable mechanoreceptor.

Under pathological conditions—such as in patients with IS—significant hemodynamic changes lead to increased PIEZO1 levels in cerebral capillary endothelial cells, resulting in calcium influx that activates downstream CaMKII and Nrf2/HO-1/NQO1 signaling pathways. This exacerbates oxidative stress and inflammatory responses in endothelial cells, thereby causing disruption of the BBB (68). In AD models, increased PIEZO1 activation is associated with reduced cerebral capillary blood flow and impaired neurovascular coupling; however, pharmacological stimulation of PIEZO1 can increase cerebral blood flow and promote hyperemia, indicating that its effects are context-dependent (69).

In summary, PIEZO1, as a key mechanosensory hub in endothelial cells, regulates BBB homeostasis by sensing mechanical changes in the ECM, thereby maintaining the autonomy of cerebral blood flow and the efficiency of waste clearance under physiological conditions; however, under pathological conditions such as ischemia and neurodegenerative diseases, its abnormal activation may exacerbate tissue damage. Future research should further clarify the activation thresholds and time windows of PIEZO1 in various CNS diseases, providing a crucial basis for the development of neurovascular protective therapies based on the regulation of mechanical signals.

## 6 Applications of the ECM-PIEZO1 axis in common CNS diseases

### 6.1 AD

In the brain tissue of AD patients and in animal models, changes in ECM components occur, primarily due to structural disruption of certain PNNs and abnormal sulfation of glycosaminoglycans (GAGs) (70), whereas PNNs are dense ECM structures rich in CSPGs that play a crucial role in maintaining synaptic stability and neuronal plasticity. On the other hand, Aβ plaques deposit extracellularly, subsequently leading to the formation of intracellular neurofibrillary tangles composed of the microtubule-associated protein tau (71, 72); these changes in ECM stiffness can affect the sensitivity of surrounding microglia, astrocytes, and neurons to mechanical stimuli in the local microenvironment, thereby triggering PIEZO1 activation.

**Figure 2:**
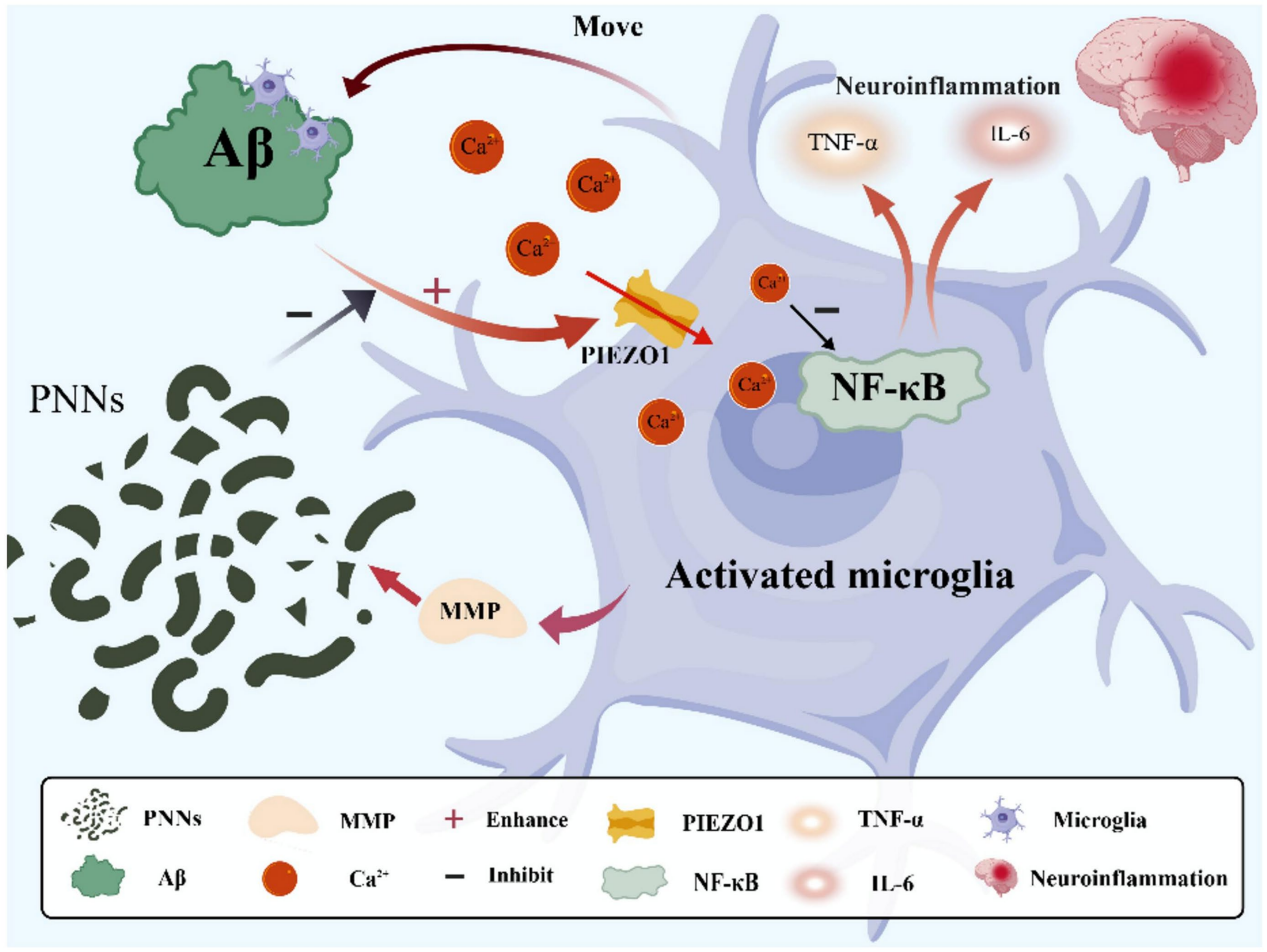
The ECM–PIEZO1 signaling axis mediates pathological vicious cycles in AD. In AD brain tissue, massive deposits of extracellular toxic A*β* plaques occur, accompanied by the disruption of CSPG-rich PNNs and changes in ECM stiffness, which in turn mechanically activate the PIEZO1 channel on microglia. PIEZO1 mediates a massive influx of Ca^2+^, which, on the one hand, promotes the directed migration of microglia toward Aβ plaques, enhancing their phagocytic capacity and lysosomal activity to clear toxic Aβ aggregates; on the other hand, elevated intracellular calcium ions inhibit the NF-κB inflammatory pathway, downregulating the release of pro-inflammatory factors such as TNF-*α* and IL-6, thereby alleviating neuroinflammation. However, activated microglia secrete large amounts of MMPs, which further degrade the PNN structure; in turn, the loss of PNN integrity weakens the microglia’s ability to respond to Aβ plaques, leading to increased plaque deposition and creating a cascading, amplifying pathological vicious cycle. PIEZO1 can serve as a key intervention target to break this vicious cycle, promote the clearance of toxic Aβ plaques by microglia, and improve cognitive impairment in AD. Created with BioGDP.com (107).

In the AD model, the presence of Aβ plaques leads to increased PIEZO1 expression in microglia, which in turn causes Ca^2+^ to enter the cells, prompting the microglia to migrate toward the plaques and enhancing their phagocytic capacity (73) and lysosomal activity to clear Aβ plaques. At the same time, PIEZO1 activation also raises intracellular calcium levels, suppresses the NF-κB inflammatory signaling pathway, and reduces the levels of pro-inflammatory cytokines such as TNF-*α* and IL-6, thereby alleviating neuroinflammation (51). In mouse studies, PIEZO1 knockout in microglia leads to exacerbated Aβ pathology and cognitive decline; however, pharmacological stimulation of PIEZO1 effectively reduces Aβ levels in the brain and improves cognitive function (73). Activated microglia, however, produce large amounts of MMP, a key factor in the breakdown and degradation of PNNs (74). At the same time, the absence of PNNs further reduces microglial responsiveness to Aβ plaques, promoting the formation of more plaques (70), thereby creating a vicious cycle. Figure 2 illustrates the disease progression mediated by the ECM-PIEZO1 axis in AD patients.

Therefore, PIEZO1 can serve as a key factor in breaking the vicious cycle between PNNs and microglia in the ECM, promoting the phagocytosis of toxic Aβ plaques by microglia and thereby improving cognitive impairment in patients with AD.

### 6.2 IS

In IS, ECM deposition and formation of fibrotic scars occur in the lesion core, constituting an important mechanical microenvironment basis for BBB injury. Disruption of BBB integrity leads to abnormally elevated permeability and subsequent cerebral edema (75–77), which represents a core event in disease pathogenesis.

The BBB primarily consists of endothelial cells, pericytes, astrocytes, and the basement membrane. Among these, the tight junctions between endothelial cells form the core structural foundation for BBB integrity, and endothelial cell damage is a key cellular event mediating the onset and progression of IS (78, 79). In IS, the loss of tight junctions between endothelial cells allows plasma proteins and peripheral immune cells to enter the brain (80). The breakdown of the BBB is initially mediated by the upregulation of pro-inflammatory factors TNF-*α* and IL-1β, triggering an inflammatory response. Under the stimulation of TNF-α and IL-6, resident astrocytes transform into reactive astrocytes; this process is typically mediated by the upregulation of Glial Fibrillary Acidic Protein (GFAP) and results in the formation of a dense glial scar around the core of the lesion. Reactive astrocytes secrete the chemokines C-C motif chemokine ligand 3 (CCL3), C-X-C motif chemokine ligand 1 (CXCL1), and C-X-C motif chemokine ligand 2 (CXCL2), thereby recruiting macrophages to the site of injury (81), While macrophages clear the site of injury, they also induce tissue remodeling accompanied by ECM deposition (82). Although this sclerotic scar limits the spread of inflammation, it also forms a physical barrier that inhibits axonal regeneration and myelin repair. During cerebral ischemia/reperfusion (I/R), reduced ATP production and intracellular acidosis jointly lead to an abnormal increase in calcium ions, which in turn induces mitochondrial dysfunction, triggering the release of pro-inflammatory factors and excessive ROS production. This, in turn, disrupts tight junction proteins and the endothelial monolayer structure (83), and further damages endothelial cells by activating multiple signaling pathways. Endothelial cells are exposed to the innermost layer of blood vessels and are directly exposed to mechanical changes in blood flow. In addition to mechanical forces from blood flow, structural changes in the BBB basement membrane-a key component of the brain tissue’s ECM-can also indirectly regulate the activation state of PIEZO1 in endothelial cells, further amplifying damage signals.

PIEZO1, as a mechanosensitive protein on endothelial cells (84), plays a key role in IS, activation of PIEZO1 triggers calcium influx, which in turn activates downstream CaMKII (85) and Nrf2/HO-1/NQO1 (68) signaling pathways, thereby exacerbating oxidative stress and inflammatory responses in endothelial cells. Studies have shown that endothelial-specific knockout of PIEZO1 reduces brain injury, improves neurological outcomes, and preserves the structural and functional integrity of the BBB (68). Thus, PIEZO1 in endothelial cells is a key mechanosensor mediating early BBB damage during IS; targeted inhibition of this channel can effectively block early brain injury, while the basement membrane, as a core component of the ECM, acts as a potential amplifying factor in this process due to its mechanical changes.

In addition to IS, for typical ischemic encephalopathies such as hypoxic–ischemic brain damage (HIBD), the influence of PIEZO1 on the mechanical microenvironment of the ECM is even more significant. a specific interactive relationship exists between the two. In the case of IS, mechanical stimuli generated by blood flow can directly activate PIEZO1 in endothelial cells, whereas in the case of HIBD, the primary cause of abnormal PIEZO1 activation is mechanical changes in the ECM. LOX expression and activity were significantly increased in HI-induced neurons and throughout the brain; this enzyme catalyzes covalent cross-linking between collagen I and III molecules in the ECM (86), making the ECM more robust, while also stimulating the production of collagen I and III (86), resulting in a stiffer matrix. Experiments have shown that the elastic modulus of brain tissue in HIBD rats is significantly increased; this abnormally elevated ECM stiffness is detected by PIEZO1 in cortical and hippocampal neurons (42), triggering a sustained influx of calcium ions. In the HI-induced neuronal model, the levels of type I and III collagen rise, accompanied by increased PIEZO1 expression and channel activation (41–43). Upon activation, PIEZO1 also reduces levels of its downstream target GPX4, leading to uncontrolled lipid peroxidation, iron accumulation, and mitochondrial damage, which in turn induce neuronal ferrocytosis. However, inhibiting either LOX or PIEZO1 can reverse ECM stiffness and calcium overload, increase GPX4 expression, and reduce ferrocytosis, while also significantly improving learning and memory impairments and synaptic plasticity deficits in HIBD rats (42).

**Figure 3:**
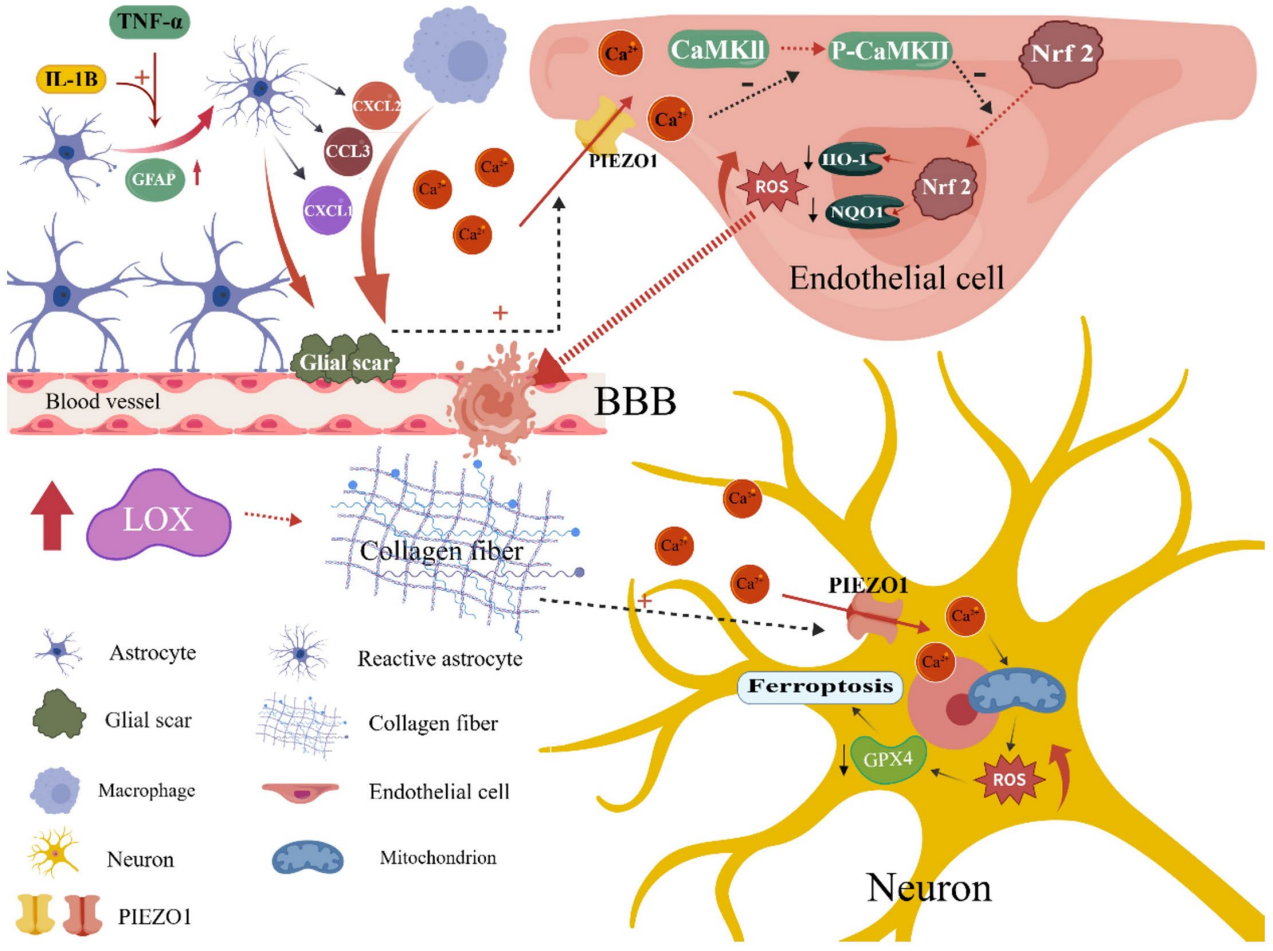
The ECM–PIEZO1 axis mediates pathological damage mechanisms in IS. Massive ECM deposition and glial scar fibrosis-induced remodeling occur in the core region of the ischemic lesion, creating a unique mechanical microenvironment that disrupts the integrity of the BBB. Pro-inflammatory factors TNF-α and IL-1β induce upregulation of GFAP expression in astrocytes, transforming them into reactive astrocytes; activated astrocytes secrete CCL3, CXCL1, CXCL2, which recruit peripheral macrophages; these infiltrating macrophages further promote tissue remodeling and collagen accumulation, exacerbating glial scar sclerosis. The stiffened ECM, combined with hemodynamic mechanical stimuli, jointly activates the mechanosensitive channel PIEZO1 on cerebral endothelial cells; PIEZO1 mediates Ca^2+^ influx, activating CaMKII and the downstream Nrf2/HO-1/NQO1 pathways, thereby exacerbating endothelial oxidative stress, disrupting tight junctions, and amplifying BBB damage. On the other hand, HI significantly upregulates the expression and activity of LOX in neurons; this enzyme mediates covalent cross-linking of collagen fibers and increases collagen synthesis, leading to an abnormal increase in ECM stiffness; mechanical signals from the highly rigid ECM continuously activate neuronal PIEZO1, triggering persistent calcium overload, downstream downregulation of GPX4 expression, mitochondrial damage, and massive accumulation of ROS, ultimately inducing neuronal ferroptosis. Specific inhibition of PIEZO1 or LOX alleviates BBB damage, reduces neuronal ferroptosis, and improves neurological deficits following ischemic brain injury. Created with BioGDP.com (107).

Overall, PIEZO1 plays a dual role in IS: it serves as a key mechanosensor that is activated when endothelial cells are damaged by blood flow-induced changes in intracellular fluid dynamics, thereby exacerbating BBB damage; simultaneously, it detects abnormal ECM accumulation within neurons and triggers a series of cascading events leading to ferroptosis. Figure 3 illustrates the specific role of the ECM-PIEZO1 axis in IS. Therefore, modulating PIEZO1 function or intervening in the ECM repair process (such as by inhibiting LOX) may represent a novel approach to preventing BBB damage, reducing neuronal death, and improving post-stroke outcomes.

### 6.3 MS

According to statistics from the www.atlasofms.org, an estimated 2.9 million people worldwide have MS, with a global prevalence of 35.9 cases per 100,000 people. It is a common neurological disorder among young adults, most frequently diagnosed between the ages of 20 and 40 (87). MS is a disease characterized by neuroinflammation, demyelination, and axonal damage, which ultimately lead to neurological dysfunction (88). Based on clinical course, MS can be classified into relapsing–remitting multiple sclerosis (RRMS), secondary progressive multiple sclerosis (SPMS), and primary progressive multiple sclerosis (PPMS); all three subtypes share inflammatory demyelination as their primary pathological feature.

Dynamic changes in the ECM occur throughout the course of MS. During the inflammatory phase, heparan sulfate (HS) in the BBB is degraded, leading to an abnormal increase in BBB permeability. Reactive astrocytes, microglia, and macrophages mediate the massive deposition of components such as fibronectin, fibrinogen, CSPG, collagen, and platelet-reactive protein (6). Among these, fibrin enters the central nervous system via the bloodstream and deposits at the center of lesions; as a pro-inflammatory factor, it amplifies the central inflammatory response (89) while simultaneously inhibiting the differentiation of OPCs and remyelination (90). Fibronectin and collagen, as key components of matrix stiffening, play a role in scar formation and provide the environment for subsequent activation of PIEZO1. As the disease enters the chronic progressive phase, demyelination becomes a key factor in its progression. CSPGs and hyaluronic acid are the primary components of the brain’s soft matrix. CSPGs are derived from reactive astrocytes and macrophages/microglia, and they migrate toward the periphery of the lesion along with immune cells (91), where they are abnormally deposited at the lesion margins, not only forming a physical barrier but also inhibiting OPCs from entering the center of the lesion for repair through chemical signaling. OPCs are mechanosensitive and can detect changes in the stiffness of their surrounding environment, allowing them to remain as precursor cells or differentiate into myelinating oligodendrocytes. A softer ECM is more conducive to OPCs differentiation. As OPCs age, the surrounding ECM environment gradually hardens (65), leading to inhibition of their proliferation, differentiation, and myelination; however, this process is reversed in a soft matrix. At the same time, the basement membrane surrounding blood vessels in MS patients is remodeled into a dense “network-like structure.” The thickened BBB impedes the exchange of substances between the brain and the periphery during the progressive phase and also blocks immune cells from entering the brain to trigger inflammatory attacks. This may be the primary reason for the reduction in inflammation during the progressive phase of MS; however, the mechanical forces exerted by perivascular cells and astrocytes also change accordingly. ECM deposition does not result in increased stiffness throughout the entire lesion center; rather, deposition is most concentrated in areas where the BBB is disrupted, resulting in a characteristic pattern where the periphery is stiffer than the central region. This stiffness gradient may serve as a key mechanical signal driving cell migration or activating PIEZO1 in peripheral cells.

The mechanosensitive ion channel PIEZO1, as a key molecule linking the mechanical microenvironment to cellular responses, has been demonstrated to be present in CNS neurons (58, 92), oligodendrocytes, T cells, microglia (93), and astrocytes in various states of activation (49, 94). It is also one of the key intermediaries through which OPCs sense matrix stiffness and exerts a negative regulatory effect on myelin formation and regeneration. Recent findings in the field of aging suggest that aged ECM and niche stiffness may impair OPCs function through signaling via the mechanosensitive ion channel PIEZO1 in OPCs (95). During the inflammatory phase, studies have found that mechanical forces contribute to optimal T-cell activation; for example, antibodies immobilized on beads induce T-cell receptor (TCR) activation more effectively than soluble antibodies (96). In the absence of PIEZO1, immobilized cross-linked antibodies fail to induce optimal TCR activation in CD4^+^ and CD8^+^ T lymphocytes (CD8^+^ T); however, chemical activation of PIEZO1 using Yoda1 can rescue this functional defect. Furthermore, inhibition of calpain impedes optimal TCR activation. In experimental autoimmune encephalomyelitis (EAE), inhibiting PIEZO1 in CD4^+^ T cells or Tregs can attenuate the inflammatory response (97, 98), which may be related to ECM matrix deposition in MS; a stiffer ECM may be a key factor in activating PIEZO1 on the surface of T cells. Upon activation, PIEZO1 inhibits the TGFβ/SMAD signaling pathway in CD4^+^ T cells and limits Treg activity (99), Since Tregs serve as a line of defense in the immune response, the suppression of their activity exacerbates the inflammatory response in MS; therefore, restoring Treg activity may be a key therapeutic target for MS, and PIEZO1 may play a pivotal role in this process. However, PIEZO1 also plays a role in promoting inflammation in macrophages. Increased ECM stiffness activates PIEZO1 channels, triggering a Ca^2+^ influx, which in turn activates the AP-1 transcription factor and upregulates Edn1 expression. Edn1 stabilizes the HIF1α protein via paracrine or autocrine mechanisms, thereby driving macrophages to adopt a sustained pro-inflammatory phenotype (M1 type) (31). PIEZO1 not only acts independently but also exerts its effects in synergy with the macrophage integrin subunit αM (CD11b) and the immune receptor Toll-like receptor 4 (TLR4). Under mechanical stimulation, PIEZO1 and CD11b exhibit dynamic expression antagonism (100). Following PIEZO1 knockout, CD11b is upregulated, while the inflammatory response is attenuated, suggesting a possible negative feedback mechanism between the two that acts synergistically in MS; under inflammatory stimulation, the assembly of the TLR4-PIEZO1 complex drives cytoskeletal remodeling and ROS bursts via the calcium/calmodulin-dependent protein kinase II-mammalian sterile 20-like kinase 1/2-Rac1 (CaMKII-Mst1/2-Rac) axis (101).

During the progressive phase, abnormalities in ECM components and changes in mechanical properties within the lesion microenvironment may continuously influence PIEZO1 signaling; PIEZO1 expression in cells such as astrocytes is upregulated in a time-dependent manner (52), contributing to the cells’ mechanical response to the abnormal matrix. Activation of PIEZO1 in axons inhibits central myelination (97), the PIEZO1 agonist Yoda1 activates PIEZO1 in axons, promoting calcium influx, which in turn triggers calcium release from the endoplasmic reticulum; this step promotes demyelination, whereas the PIEZO1 inhibitor GsMTx4 blocks PIEZO1 activity and prevents demyelination (102). Not only that, but deposited ECM may be key to activating PIEZO1, and the activation of PIEZO1 inhibits axonal regeneration. Upon axonal injury, PIEZO1 is recruited to the growth cone and activated, where it inhibits axonal regeneration via the CaMKII-Nos-PKG pathway, creating a vicious cycle (103).

**Figure 4:**
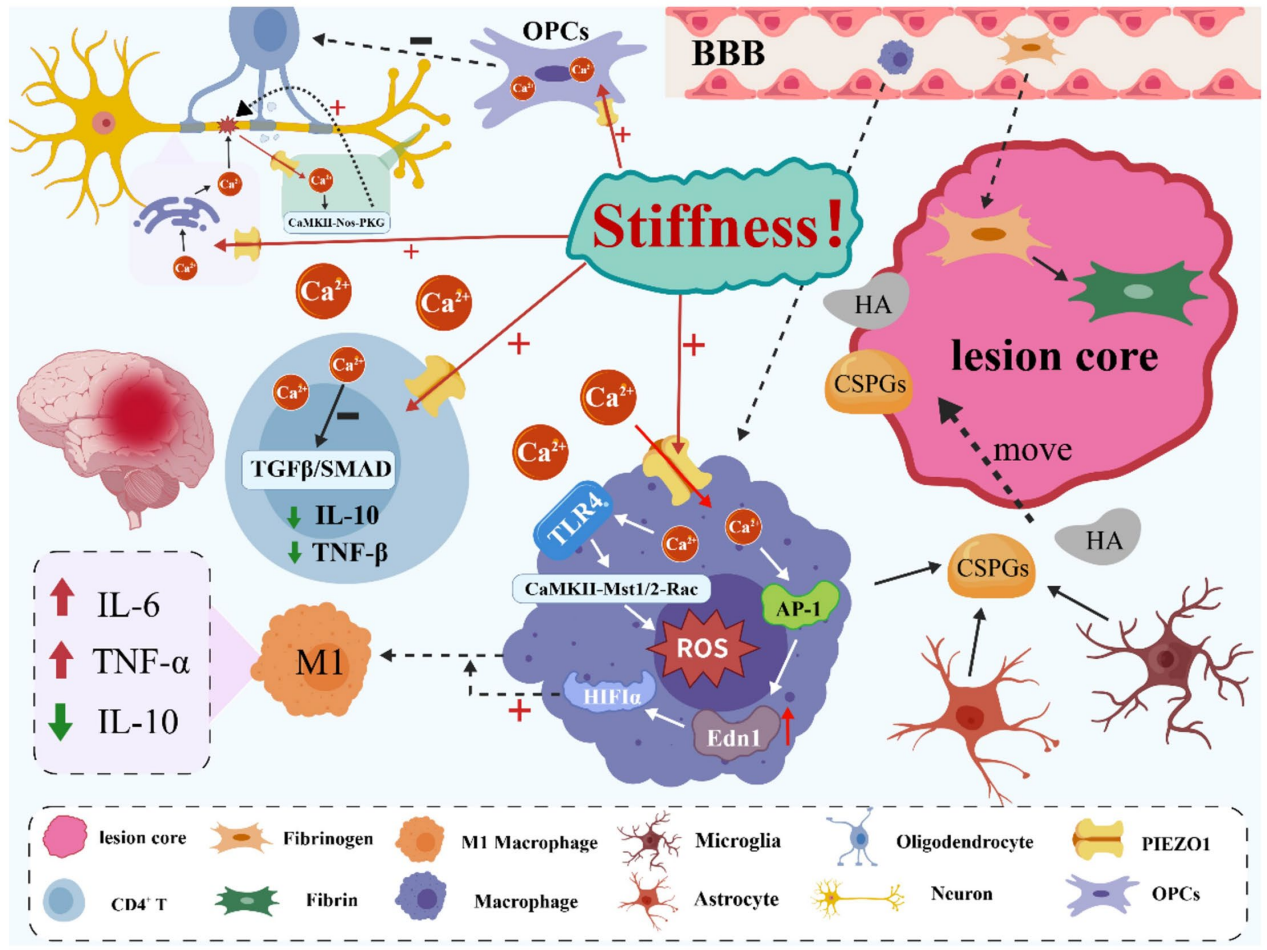
Mechanisms of the ECM-PIEZO1 mechanical signal transduction axis in the pathological progression of MS. Breaches in the BBB at MS lesions lead to abnormal ECM deposition and increased tissue stiffness, which in turn activate the mechanosensitive PIEZO1 channel in various types of central nervous system cells. Within macrophages, PIEZO1 acts in concert with TLR4 to mediate Ca^2+^ influx and massive ROS production, thereby activating the activator protein 1/endothelin-1/hypoxia-inducible factor 1α (AP-1/Edn1/HIF-1α) pathway, thereby maintaining the pro-inflammatory M1 phenotype of macrophages; in CD4^+^ T cells, PIEZO1 inhibits the TGFβ/SMAD pathway, impairs Treg function, and exacerbates neuroinflammation. The sclerotic matrix simultaneously activates PIEZO1 in OPCs, neurons, and astrocytes, inhibiting OPCs differentiation, promoting demyelination, and blocking axonal regeneration via the CaMKII-Nos-PKG pathway, thereby creating a pathological vicious cycle. CSPG and HA secreted by activated glial cells accumulate at the lesion margins, further impeding myelin repair. As a central mechanotransduction molecule, PIEZO1 senses mechanical changes in the ECM and mediates the entire process of neuroinflammation, impaired myelin regeneration, and matrix remodeling in MS. Created with BioGDP.com (107).

In summary, by sensing mechanical and compositional changes in the ECM, PIEZO1 bidirectionally regulates neuroinflammation, myelin regeneration defects, and matrix remodeling at different stages of MS, serving as a key mechanical transduction node linking the ECM microenvironment to cellular function. The specific mechanism is illustrated in Figure 4.

## 7 Conclusions and observations

This review focuses on the ECM-PIEZO1 axis, with the ECM as the upstream factor and PIEZO1 as the downstream factor. It discusses the major changes in the ECM within the CNS and the downstream pathways that trigger PIEZO1 responses, and explores the applications of the ECM-PIEZO1 axis in CNS diseases. Unlike previous reviews, which have largely focused on PIEZO1’s downstream effects, this paper not only systematically summarizes PIEZO1-mediated mechanosensitive responses but also emphasizes the upstream ECM remodeling characteristics and molecular triggers that drive abnormal PIEZO1 activation.

First, the unique triple-helix structure of PIEZO1 serves as the key structural basis for its sensing of membrane tension and curvature. In environments with high matrix stiffness, PIEZO1 channels are activated primarily by sensing changes in membrane tension, whereas in softer matrices (such as the normal brain), integrins are required to amplify mechanical signals (27, 28). Second, multiple mechanical signals from the ECM serve as upstream mechanisms regulating PIEZO1 activation, primarily including matrix stiffness, ECM stiffening induced by LOX-mediated collagen cross-linking, inflammation-mediated abnormal ECM deposition, and tissue viscoelasticity.

PIEZO1 is widely expressed in CNS cells, but its functions vary; the causal differences between stimulation and response, as well as the upstream-downstream signaling relationships, provide a reasonable explanation for this controversial phenomenon. In neurons and oligodendrocytes, the primary roles of PIEZO1 are to regulate axon and myelin formation, respectively, whereas in microglia and astrocytes, PIEZO1 focuses on regulating inflammatory pathways; however, its function exhibits distinct cell-specificity. Activation of PIEZO1 in microglia can inhibit NF-κB signaling, thereby attenuating the inflammatory response (51); however, in astrocytes, PIEZO1 expression is positively correlated with the levels of pro-inflammatory factors TNF-*α*, IL-1β, and NF-κB (52). These seemingly contradictory results are not inconsistent; the key lies in the fundamental difference in the causal sequence between the two cell types. In microglia, PIEZO1 acts as an upstream molecule to actively suppress inflammation, whereas in astrocytes, elevated PIEZO1 expression is a downstream feedback response to TNF-α-induced inflammatory stimulation (52). This difference in causality may be the key factor underlying the bidirectional nature of PIEZO1’s regulation of inflammation.

The activation and inhibition of PIEZO1 are not strictly positive or negative; their effects may differ under normal and pathological conditions. In normal mouse models, activation of PIEZO1 on astrocytes promotes the release of ATP and maintains connections with neurons and microglia, whereas its absence impairs adult neurogenesis (50). However, following IS, downregulation of PIEZO1 in astrocytes actually promotes neurogenesis and motor function recovery (61), and it also regulates astrocyte proliferation by influencing YAP-related proteins (62). Abnormal ECM deposition is common in disease processes such as valvular calcification (43), intervertebral disc degeneration (104), and fibrosis (105), and constitutes the pathological basis for disease onset; therefore, inhibiting PIEZO1 may play a beneficial role in these conditions. In contrast, since the breakdown of ECM structure is a key pathogenic factor in AD (73), inhibiting PIEZO1 in this context would have adverse effects. In summary, the bidirectional regulatory role of PIEZO1 in disease is primarily determined by changes in the mechanical microenvironment triggered by upstream ECM remodeling; this also explains why PIEZO1 functions differently under various pathological conditions.

Although PIEZO1 acts as a sensor for ECM stiffness, it can also influence the ECM in turn, creating a bidirectional regulatory relationship. PIEZO1 in neurons exerts a bidirectional regulatory effect on axons: matrix stiffness can regulate axonal regeneration via PIEZO1 (53), and conversely, PIEZO1 can also remodel the matrix by altering axonal behavior (56); In AD, while PIEZO1 in microglia plays a role in clearing Aβ and providing anti-inflammatory protection, it also destroys PNNs by secreting MMPs, thereby exacerbating plaque deposition; a similar phenomenon has been observed in studies of squamous cell carcinoma, where increased ECM stiffness triggers PIEZO1 activation in tumor cells, and tumor cells, through interactions with fibroblasts, promote ECM remodeling and further increase the already high matrix stiffness (106). This suggests that future intervention strategies, in addition to directly targeting PIEZO1, could also exert regulatory effects by modulating the mechanical microenvironment through ECM regulation.

Therefore, future research should not only focus on the specific functions of PIEZO1 in different cell types but also emphasize the differences in its roles under physiological and pathological conditions. In particular, during the progression of IS, the structure and components of the ECM undergo dynamic changes, suggesting that the function of PIEZO1 may undergo stage-specific transformations as the disease progresses. Consequently, simply investigating the expression and function of PIEZO1 alone cannot fully elucidate its mechanism of action; future research must further focus on the upstream factors that regulate the activation or inhibition of PIEZO1. As a central upstream regulator of mechanosensory signaling, ECM-mediated changes in the mechanical microenvironment serve as the key foundation for regulating the functional plasticity of PIEZO1 and will become a priority area for future research in this field.

## Glossary

AD: Alzheimer’s disease
Akt: protein kinase B
AMPK: adenosine monophosphate-activated protein kinase
Anchor: anchor domain
AP-1: activator protein-1
ATF4: activating transcription factor 4
ATP: Adenosine Triphosphate
A*β*: Amyloid β-protein
BBB: Blood–Brain Barrier
Beam: intracellular lever beam domain
Blade: transembrane helical blade domain
calpain: calcium-activated neutral protease
CaMKII: calcium/calmodulin-dependent protein kinase II
Cap: extracellular cap domain
CCL3: C-C motif chemokine ligand 3
CD11b: integrin subunit αM
CD4 + T: CD4^+^T lymphocyte
CD8^+^ T: CD8^+^ T lymphocyte
CHOP: C/EBP homologous protein
CNS: Central Nervous System
CSPG: Chondroitin sulfate proteoglycan
CTD: C-terminal cytoplasmic domain
CXCL1: C-X-C motif chemokine ligand 1
CXCL2: C-X-C motif chemokine ligand 2
EAE: Experimental Autoimmune Encephalomyelitis
ECM: Extracellular matrix
Edn1: Endothelin-1
FA: Focal Adhesion
FAK: focal adhesion kinase
GAG: glycosaminoglycans
GFAP: Glial Fibrillary Acidic Protein
GPX4: Glutathione peroxidase 4
GsMTx4: Grammostola spatulata mechanotoxin 4
HA: Hyaluronic acid
HI: Hypoxia-Ischemia
HIBD: Hypoxic–Ischemic Brain Damage
HIF1α: hypoxia-inducible factor 1α
HO-1: heme oxygenase-1
HS: Heparan Sulfate
I/R: Ischemia/Reperfusion
IH: inner helix
IL-1β: Interleukin-1β
IL-6: Interleukin-6
IRE1: inositol-requiring enzyme 1α
IS: Ischemic Stroke
ITGα5: integrin subunit α5
LOX: Lysyl Oxidase
LPS: lipopolysaccharide
LTP: Long-term potentiation
MMP13: matrix metalloproteinase 13
MS: Multiple Sclerosis
Mst1/2-Rac: mammalian sterile 20-like kinase 1/2–Rac1
NF-κB: Nuclear Factor-kappa B
Nos: nitric oxide synthase
NQO1: NAD(P)H quinone dehydrogenase 1
Nrf2: nuclear factor erythroid 2–related factor 2
NVU: Neurovascular Unit
OH: outer helix
ONH: Optic Nerve Head
OPCs: Oligodendrocyte Precursor Cells
OXPHOS: oxidative phosphorylation
p38 MAPK: p38 mitogen-activated protein kinase
PERK: protein kinase RNA-like endoplasmic reticulum kinase
PI3K: phosphatidylinositol 3-kinase
PiCS: Piezo1-induced Cytoskeletal Stiffening
PKG: protein kinase G
PNNs: Perineuronal Nets
PPMS: Primary Progressive Multiple Sclerosis
RHOA: Ras homolog family member A
ROCK: Rho-associated coiled-coil-containing protein kinase
ROS: Reactive Oxygen Species
RRMS: Relapsing–Remitting Multiple Sclerosis
SDHA: succinate dehydrogenase complex flavoprotein subunit A
Sema3A: semaphorin 3A
siRNA: small interfering RNA
SIRT3: sirtuin 3
Slit1: slit guidance ligand 1
SMAD: Sma-and Mad-related protein
SPMS: Secondary Progressive Multiple Sclerosis
TAZ: transcriptional coactivator with PDZ-binding motif
TCR: T-Cell Receptor
TGF*β*: transforming growth factor-β
Th9: T helper 9 cell
TLR4: Toll-like Receptor 4
TM: transmembrane helix
TNF-*α*: Tumor necrosis factor-α
TNS1: tensin 1
Treg: Regulatory T cells
ULK1: unc-51-like autophagy activating kinase 1
Wnt7b: Wnt family member 7B
YAP: Yes-associated protein
